# 3,5-Diiodo-L-Thyronine Activates Brown Adipose Tissue Thermogenesis in Hypothyroid Rats

**DOI:** 10.1371/journal.pone.0116498

**Published:** 2015-02-06

**Authors:** Assunta Lombardi, Rosalba Senese, Rita De Matteis, Rosa Anna Busiello, Federica Cioffi, Fernando Goglia, Antonia Lanni

**Affiliations:** 1 Dipartimento di Biologia, Università degli Studi di Napoli “Federico II”, Napoli, Italy; 2 Dipartimento di Scienze e Tecnologie Ambientali, Biologiche e Farmaceutiche, Seconda Università degli Studi di Napoli, Caserta, Italy; 3 Dipartimento di Scienze Biomolecolari, Sezione di Scienze Motorie e della Salute Università degli Studi di Urbino “Carlo Bo”, Urbino, Italy; 4 Dipartimento di Scienze e Tecnologie, Università degli Studi del Sannio, Benevento, Italy; University of Santiago de Compostela School of Medicine—CIMUS, SPAIN

## Abstract

3,5-diiodo-l-thyronine (T2), a thyroid hormone derivative, is capable of increasing energy expenditure, as well as preventing high fat diet-induced overweight and related metabolic dysfunction. Most studies to date on T2 have been carried out on liver and skeletal muscle. Considering the role of brown adipose tissue (BAT) in energy and metabolic homeostasis, we explored whether T2 could activate BAT thermogenesis. Using euthyroid, hypothyroid, and T2-treated hypothyroid rats (all maintained at thermoneutrality) in morphological and functional studies, we found that hypothyroidism suppresses the maximal oxidative capacity of BAT and thermogenesis, as revealed by reduced mitochondrial content and respiration, enlarged cells and lipid droplets, and increased number of unilocular cells within the tissue. In vivo administration of T2 to hypothyroid rats activated BAT thermogenesis and increased the sympathetic innervation and vascularization of tissue. Likewise, T2 increased BAT oxidative capacity *in vitro* when added to BAT homogenates from hypothyroid rats. *In vivo* administration of T2 to hypothyroid rats enhanced mitochondrial respiration. Moreover, UCP1 seems to be a molecular determinant underlying the effect of T2 on mitochondrial thermogenesis. In fact, inhibition of mitochondrial respiration by GDP and its reactivation by fatty acids were greater in mitochondria from T2-treated hypothyroid rats than untreated hypothyroid rats. *In vivo* administration of T2 led to an increase in PGC-1α protein levels in nuclei (transient) and mitochondria (longer lasting), suggesting a coordinate effect of T2 in these organelles that ultimately promotes net activation of mitochondrial biogenesis and BAT thermogenesis. The effect of T2 on PGC-1α is similar to that elicited by triiodothyronine. As a whole, the data reported here indicate T2 is a thyroid hormone derivative able to activate BAT thermogenesis.

## INTRODUCTION

Brown adipose tissue (BAT) is a thermogenic tissue specializing in the conversion of lipids into heat. It is characterized by an extensive network of blood capillaries and is highly innervated by noradrenergic fibers. Brown adipocytes contain triglycerides within multiple small vacuoles, as well as numerous mitochondria [1].

Upon stimulation by sympathetic nervous input, a catabolic program is induced in BAT that involves acute breakdown of cellular triglyceride stores and transient activation of peroxisome proliferator-activated receptor gamma co-activator 1α (PGC-1α) [2,3] This results in efficient uptake and subsequent utilization of fuels for the production of heat. Heat production is due to a protein found almost exclusively in brown adipocytes, uncoupling protein-1 (UCP1). UCP1 is able to uncouple electron transport from ATP production in the mitochondrial respiratory chain by allowing protons to leak back across the inner mitochondrial membrane [4,5]. The resulting decrease in proton electrochemical gradient allows substrate oxidation to occur, by-passing capture of some of the useful energy via synthesis of ATP. The energy-dissipating capacity of BAT via UCP1 is significant and has the potential to increase energy expenditure of the whole animal by up to 20% [6].

UCP1-deficient mice are cold-sensitive [7], and when housed at thermoneutrality (i.e., in the absence of cold-induced thermogenesis), they exhibit an increased susceptibility to diet-induced obesity [8]. Conversely, over-expression of UCP1 or powerful activation of BAT thermogenesis prevents the development of obesity [9]. To sustain its thermogenic function, BAT initially uses stored lipids as a substrate, but for longer-lasting thermogenesis, it requires supplementary energy supplies. Indeed, when active, BAT is able to take up (and dispose of) large quantities of lipids, glucose [10,11], and lactate [12] from the circulation, which can have a significant effect on the level of both triglycerides and glucose in the blood [11,13]. This indicates that BAT plays a significant role in metabolic homeostasis.

Previously, the widely held view was that human BAT disappears rapidly after birth and is no longer present in adults. In recent years, however, positron emission tomography has revealed that: i) metabolically active BAT is present in defined regions and is scattered throughout the white adipose tissue (WAT) of adult humans [14–16], and ii) the amount of BAT detectable in adult humans positively correlates with resting metabolic rate and inversely with body mass index and fat mass [17]. Therefore, BAT is emerging as a potential target for the treatment of human obesity and related diseases [11,13,18]. Identification of molecules able to initiate and/or maintain thermogenic activation of brown adipose cells is important for attempts to devise therapeutic strategies to counteract obesity and related pathologies (e.g., insulin resistance and hyperlipidemia). Among the endocrine factors able to activate BAT, thyroid hormone triiodothyronine (T3) plays a major role [5,19]. Indeed, BAT thermogenesis significantly contributes to thyroid regulation of energy balance and explains the important role played by T3 in energy homoeostasis and adaptation to cold [5,20].

In recent years, accumulating evidence has indicated that among thyroid hormone derivatives, the biological activity of 3,5-diiodo-l-thyronine (T2) is of particular interest [21]. The effects induced by T2 at the mitochondrial level in liver and skeletal muscle are: i) a rapid increase in respiration rate [22,23] that is independent of transcription or translation mechanisms [23–25], ii) an increase in the fatty acid oxidation rate [26–28], and iii) activation of thermogenic processes [23, 26–28]. At the level of the whole animal, T2 rapidly stimulates the metabolic rate in rats [29] and prevents them from developing high-fat diet-induced overweight [28, 30]. In addition, administration of T2 ameliorates the occurrence of metabolic diseases associated with the prolonged intake of a high-fat diet (e.g., liver steatosis, hypertriglyceridemia, insulin resistance) [28,30–32]. Similarly, in humans, a case report study showed that T2 is able to increase the resting metabolic rate and reduce body weight without undesirable side effects [33].

Most studies on T2 metabolic effects so far have focused on liver and skeletal muscle. Moreover, studies on the ability of T2 to activate BAT thermogenesis have been limited to its capacity to stimulate BAT cytochrome oxidase (COX) activity when administered to hypothyroid rats either maintained at thermoneutrality [34] or cold-exposed [35]. In view of the significant impact of BAT on whole-body metabolism both in rodents and possibly adult humans, we investigated whether BAT thermogenesis could be activated by T2 using morphological and functional analyses. We also investigated the possibility that some of the molecular events underlying the T2 effect on BAT thermogenesis are also affected by T3. Experiments involved the use of hypothyroid rats, in which BAT thermogenesis and oxidative capacity are reduced [34,35], maintained at thermoneutrality so as to minimize the effects of increased sympathetic tone. To induce severe hypothyroidism, while inhibiting all three known types of iodothyronine deiodinase enzymes, we combined treatment with propylthiouracil and iopanoic acid [29,36]. This allowed us to attribute observed effects to the injected iodothyronine(s) rather than to any of their deiodinated products, and exclude the possibility that any observed effect of T2 involving activation of BAT thermogenesis might be mediated by local formation of T3.

In the present study, by performing morphological and functional analysis, we showed the ability of T2 to activate BAT thermogenesis.

## METHODS

All chemicals were purchased from Sigma-Aldrich (St. Louis, MO, USA) unless specified otherwise.

### Animals

Male Wistar rats (275–300 g) were obtained from Harlan Laboratories (Italy) and housed one per cage in a 28°C temperature-controlled room under a 12:12-h light-dark cycle; commercial mash and water were available *ad libitum*. Four experimental groups of six rats each were used: 1) group “Eu,” consisting of vehicle-injected euthyroid rats; 2) group “Hypo,” consisting of hypothyroid rats, wherein hypothyroidism was induced by intraperitoneal (i.p.) injection of 1 mg/100 g body weight propylthiouracil for 4 weeks together with a weekly i.p. injection of 6 mg/100 g body weight iopanoic acid [27,32,34]; 3) group “Hypo+T2,” consisting of Hypo rats that received either daily injection of T2 (>99% pure; 25 μmg/100 g body weight; “Hypo+T2”) in the final week of treatment or 1 h (“Hypo+T21h”) before being euthanized; and 4) group “Hypo+T3,” consisting of Hypo rats that received either daily injection of T3 (>95% pure; 15 μg/100 g body weight; “Hypo+T3”) in the final week of treatment or a single injection of T3 (25 μg/100 g body weight) 1 h before being euthanized (“Hypo+T31h”).

At the end of treatments, rats were anesthetized by i.p. injection of chloral hydrate (40 mg/100 body weight) and killed by decapitation. Blood was collected and intrascapular BAT tissues were excised, weighed, and immediately processed for mitochondrial isolation and histological analysis or frozen in liquid nitrogen for later use.

This study was carried out in strict accordance with recommendations in the Guide for the Care and Use of Laboratory Animals of the National Institutes of Health (USA). All animal protocols were approved by the Committee on the Ethics of Animal Experiments of the University of Napoli Federico II (Italy) and the Italian Minister of Health (Permit Number: 2011/0041469). Every effort was made to minimize animal pain and suffering.

### Energy expenditure

The 24 h prior to the end of each treatment, rat energy expenditure was measured by indirect calorimetry, as well as evaluation of oxygen consumption (VO_2_), carbon dioxide production (CO_2_), and respiratory quotient (VCO_2_/VO_2_). Measurements were made using a four-chamber, indirect, open-circuit calorimeter Oxymax system (Panlab, Cornella, Barcelona, Spain) with one rat per chamber. After a 1-h period of adaptation to the metabolic chamber, VO_2_ and VCO_2_ were measured in individual rats at 15-min intervals for 4 h. Energy expenditure was then calculated in Kcal/day. Kg^0.75^

### Determination of COX and citrate synthase activity in BAT homogenates

COX activity was determined polarographically at 25°C using a Clark-type oxygen electrode, as described by Lanni *et al*. [34]. This procedure required 1.5 ml of reaction medium containing 30 μM cytochrome *c*, 4 μM rotenone, 0.5 mM dinitrophenol, 10 mm sodium malonate, and 75 mm HEPES buffer (pH 7.4). Samples of intrascapular BAT were finely minced, diluted 1:10 (w/v), in modified Chappel-Perry medium (1 mM ATP, 50 mM HEPES buffer [pH 7.4], 100 mM KCl, 5 mM MgC1_2_, 1 mM EDTA, and 5 mM EGTA), and homogenized in the same medium. The homogenate was then diluted 1:2 (v/v) in Chappel and Perry medium supplemented with lubrol (10 mg / ml) to unmask the enzyme activity of the tissue. Samples were then left on ice for 30 min. To examine the ability of T2 to directly stimulate COX activity, various amounts of iodothyronine (≤10–5M) were pre-incubated *in vitro* for 30 min with homogenate from the BAT of Hypo rats.

Citrate synthase activity was determined spectrophotometrically at 30°C according to the method of Srere *et al*. [37]. The principle of this assay was to initiate the reaction of acetyl-CoA with oxaloacetic acid and link the release of free CoA-SH to a colorimetric reagent, dithionitrobenzoic acid (DTNB; acetyl-CoA + OAA + H2O ↔ citrate + CoA-SH, then CoA-SH + DTNB → TNB + CoA-S-S-TNB). The readout product used was TNB, which can be quantified by its intense absorption at 412 nm. BAT homogenates were frozen under liquid nitrogen and thawed four times to disrupt the mitochondrial membrane and expose citrate synthase.

### Determination of mitochondrial respiration rate

Briefly, immediately after excision of intrascapular BAT, fragments of this tissue were deprived of all visible WAT contamination, immersed in ice-cold isolation buffer (220 mM mannitol, 70 mM sucrose, 20 mM Tris-HCl, 1 mM EDTA, 5 mM EGTA, and 1% fatty acid-free bovine serum albumin [BSA]; pH 7.4), and then homogenized in a Potter-Elvehjem homogenizer. To remove fat, the homogenate was centrifuged at 8000 *x g* for 10 min at 4°C. Floating fat was removed, the pellet resuspended in the supernatant, and an aliquot of this fat-deprived homogenate was stored a 4°C until needed for respiratory measurements. The remainder of the homogenate was centrifuged at 700 *x g* for 10 min, and the resulting supernatant was centrifuged at 3000 *x g* to obtain a mitochondrial pellet. Mitochondrial pellets were washed twice and resuspended in a minimal volume of isolation medium and kept on ice. Protein content was determined by the method of Harthree [38].

Mitochondrial respiration was evaluated both in fat-deprived homogenate (0.3 mg protein) and in isolated mitochondria (0.1 mg protein) and assessed polarographically using a Clark-type electrode at 30°C in a final volume of 0.5 ml of 80 mM KCl, 50 mM HEPES (pH 7.0), 1 mM EGTA, 5 mM K_2_HPO_4_, and 1% fatty acid-free BSA (w/v). Respiration was initiated by addition of 5 mM α-glycerophosphate. To assess the involvement of UCP1 variations in the respiratory rate of isolated mitochondria, 1 mM guanosine diphosphate (GDP) and increasing amounts of arachidonic acid (≤180 μM) were sequentially added to substrate-energized mitochondria.

### Preparation of intrascapular BAT lysate for Western blotting

For Western blotting analysis, frozen intrascapular BAT was homogenized using an Ultra-turrax homogenizer in RIPA buffer (150 mM NaCl, 1.0% Triton X-100, 0.5% sodium deoxycholate, 0.1% SDS, 50 mM Tris, pH 8.0) supplemented with 1 mM Na_3_VO_4_, 1 mM PMSF, and 1 mg/ml leupeptin. The BAT homogenate was left on ice for 1 h, during which time it was shaken every 10 min. The lysate was then ultracentrifuged at 86,000 *x g* for 10 min at 4°C.

In order to evaluate UCP1 and voltage-dependent anion channel-1 (VDAC1) levels, intrascapular BAT lysate (15 μg) was resuspended in SDS loading buffer, followed by heating for 2 min at 90°C. Then, protein from a single rat was loaded in each lane and electrophoresed on a 13% SDS-PAGE gel. UCP1 and VDAC1 levels were detected in BAT lysates using anti-UCP1 (AB1426; Millipore) and anti-VDAC1 (114187; Gene Tex) primary antibodies, respectively. Equal loading was verified using α-tubulin as a control (Ab4074A; Abcam, Cambridge, UK).

PGC-1α protein levels were detected in nuclei- and mitochondrial-rich fractions. To obtain these enriched fractions, 100 mg of BAT tissue was homogenized in 1 ml isolation buffer (described above, deprived of BSA and supplemented with protease inhibitor cocktail), and centrifuged at 700 *x g* to yield pellet-1 and supernatant-1. Pellet-1, which represents the nuclei-rich fraction, was washed twice, resuspended in RIPA buffer, incubated on ice for 1 h, and then centrifuged at 20,000 *x g* for 20 min; the supernatant was used for Western blot analysis.

Supernatant-1 was also centrifuged at 700 *x g*; the resulting pellet was discarded and the supernatant further centrifuged at 8000 *x g*. This pellet, representing the mitochondria-rich fraction, was washed twice, resuspended in RIPA buffer, incubated on ice for 1 h, and centrifuged at 20,000 *x g* for 20 min; the supernatant was used for Western blot analysis.

In order to evaluate PGC1- α in in nuclei- and mitochondrial-rich fractions 15 μg proteins were resuspended in SDS loading buffer, followed by heating for 2 min at 90°C. Then, protein from a single rat was loaded in each lane and electrophoresed on a 8% SDS-PAGE gel. PGC1- α was detected using anti- PGC1- α (Millipore AB3242), VDAC1 and COX IV are typically used as mitochondrial loading controls, they are regulated by thyroid state and therefore, cannot be used as loading controls in this study. Because α-tubulin is an inherent component of mitochondrial membranes [39], it was used instead as a mitochondrial fraction loading control.

### Histological analysis and immunohistochemistry

Intrascapular BAT was dissected and fixed by immersion in 4% formaldehyde in 0.1 M phosphate buffer overnight at 4°C. Samples were then dehydrated in ethanol, cleared, and finally embedded in paraffin blocks. Tissues were cut into serial 3-μm-thick sections and either stained with hematoxylin-eosin for morphological investigations or processed for immunohistochemical studies. For immunohistochemistry, tissues were incubated with a primary polyclonal anti-rat UCP1 antibody raised in sheep (1:8000 dilution; kindly provided by D. Ricquier, Paris, France), anti-COX IV [1:250 dilution; MA5-15078; (Thermo Scientific, USA), Euroclone, Pero, Italy], and anti-tyrosine hydroxylase (TH) antibodies (diluted 1:700; AB10312; Immunological Sciences, Rome, Italy). Immunoreactivity was assessed according to the avidin-biotin-peroxidase method with the aid of an ABC Vectastain-Elite Kit (Vector Labs), and peroxidase activity was revealed by the use of 3,3’-diaminobenzidine tetrahydrochloride as the chromogen. Sections were counterstained with hematoxylin to reveal nuclei and mounted in Eukitt (Kindler, Freiburg, Germany). Omission of the primary antibody served as the negative control. Vascularization of intrascapular BAT was assessed by use of the biotinylated forms of the lectin *Bandeiraea simplicifolia* agglutinin 1(BS-1; L3759), an immunohistochemical marker of endothelial cells, as previously described [12]. For detection, a Vectastain ABC-AP Kit (Vector Lab) was employed, followed by development using a Fucsin + Substrate Chromogen System (Dako) and counterstaining with hematoxylin.

### Morphometric analysis

Morphometric evaluations were performed on BAT from Eu, Hypo, and Hypo+T2 rats (each n = 3) with three different hematoxylin-eosin slides (sectioned every 300 μm) analyzed for each animal. Adipocytes were identified as unilocular (UL, containing a single large vacuole), paucilocular (PL, exhibiting a large vacuole surrounded by at least five small lipid droplets), or multilocular (ML, containing more than five small homogeneous lipid droplets) [40]. For each animal, unilocular, paucilocular, and multilocular adipocytes were counted in 25 randomly selected fields. The density of each cell-type was expressed as a percentage of the total number of adipocytes counted (n = 3 per group and at least 1500 adipocytes analyzed for each experimental condition). A Nikon Eclipse 80i light microscope (Nikon Instruments, Italy) fitted with a 100X oil immersion objective (1250X final magnification) was used.

Multilocular adipocyte area and lipid droplet size were determined using an ACT-2U image analyzer linked to a Sony camera (n = 3 animals per group and at least 30 adipocytes per animal were analyzed). TH immunoreactive parenchymal nerve fibers (i.e., fibers closely associated with brown adipocytes) were counted in BAT, as previously described [41]. Twenty randomly selected multilocular areas were studied in the BAT depot of each animal. The density of TH immunoreactive fibers was expressed as the number of fibers per adipocyte. Quantification of BS-1-positive capillaries per adipocyte was obtained by counting 20 different fields from each animal; results were expressed as the number of BS-1-positive capillaries per adipocyte.

### Statistical analysis

Data were analyzed by one-way ANOVA followed by a Student-Newman-Keuls post-test, or Student’s t-test to assess any statistical differences between experimental groups. Differences were considered statistically significant when P<0.05.

## RESULTS

### Metabolic parameters

**Figure 1:**
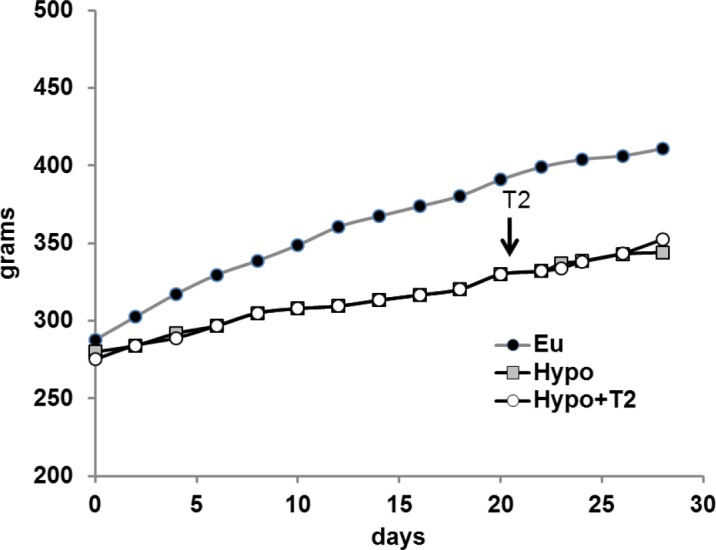
Growth curve of euthyroid (Eu), hypothyroid (Hypo), and hypothyroid rats treated with T2 (Hypo+T2). The arrows indicate initiation of T2 treatment. Although the standard error for each data point is less than 2%, these bars were omitted for a better representation.

To test the efficacy of treatment with propylthiouracil and iopanoic acid, we monitored rat food intake, body weight, and thyroid hormone levels (i.e., parameters known to be significantly reduced in hypothyroid conditions) [Table 1]. As expected, propylthiouracil- and iopanoic acid-treated rats showed a decrease in food intake (−20%Table 1) and body weight (−50%; Fig. 1). T3 and T4 serum levels were significantly reduced compared to Eu controls, indicating propylthiouracil and iopanoic acid treatment was effective at inducing severe hypothyroidism. On the other hand, administration of T2 to Hypo rats did not affect serum T3 and T4 levels (Table 1).

**Table 1:** Effect of hypothyroidism and T2 administration to hypothyroid rats on rat resting energy expenditure and on the contribution of different tissues to rat total body weight.

	**Eu**	**Hypo**	**H+T2 1w**
Total T3 serum level (nM)	0.88 ± 0.06	0.20 ± 0.04*	0.23 ± 0.04*
Total T4 serum level (nM)	55.6 ± 3.0	7.1 ± 0.9*	7.3 ± 0.9*
Energy expenditure at rest Kcal produced/day·Kg^0.75^	73.2± 4.2	52.0 ± 3.5 *	61.2±2.2*,#
Food intake (gr)	444±19	317±7*	345±15*
I-BAT/b.w. ×100	0.150 ± 0.008	0.131 ± 0.004*	0.171±0.009#
WAT(visceral depots)/b.w. × 100	4.6 ± 0.3	4.0 ± 0.2*	3.6 ± 0.3*
Liver /b.w. × 100	2.97 ± 0.12	2.72 ±0.01	2.79 ± 0.01
Gastrocnemius/b.w. × 100	1.12 ± 0.06	1.15 ±0.03	1.10 ± 0.05
Heart weight/b.w. × 100	0.27 ± 0.02	0.30 ±0.01	0.29 ± 0.01

Value are mean ± ES of 6 different rats.

*P<0,05 vs Euthyroid

#P<0,05 vs Hypothyroid rats.

To test the efficacy of T2 treatment, we evaluated rat resting energy expenditure. Hypothyroidism induced a significant decrease in resting energy expenditure (−26%), while T2 administration to Hypo rats significantly increased this expenditure (+13% versus Hypo), indicating the efficacy of T2 administration (Table 1). The contribution of BAT tissue to total body weight was decreased in the Hypo condition (−14% versus Eu), whereas T2 administration significantly increased total body weight in hypothyroid rats (+31% versus Hypo). Chronic T2 administration, however, did not significantly affect the contribution of other tissues to total body weight (Table 1), despite a tendency towards decreased visceral adiposity.

### BAT morphology, sympathetic innervation, and vascularization

**Figure 2:**
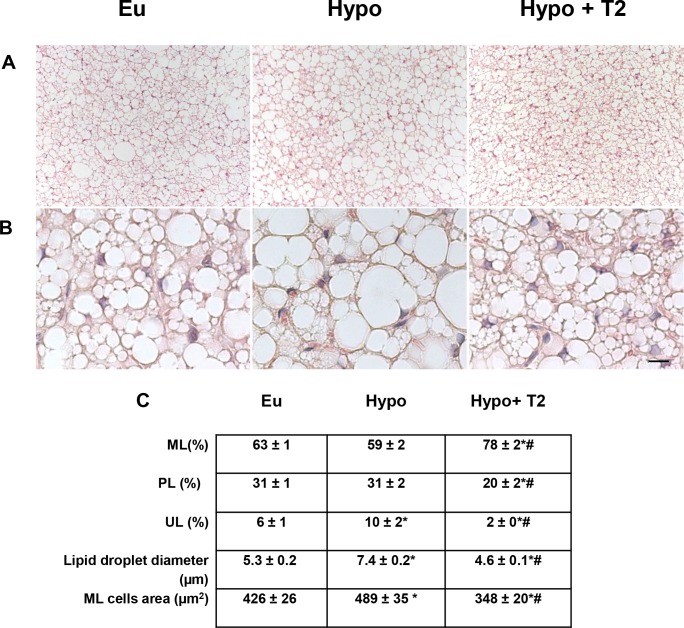
Effects of hypothyroidism and T2 administration to Hypo rats on BAT morphology. (A) Representative tissue sections showing different cellular shapes within BAT parenchyma, and (B) the different distribution of lipid droplets inside the multilocular (ML) adipocytes. Scale bars: 60 μm in (A); 10 μm in (B). (C) Quantification of ML, paucilocular (PL), and unilocular (UL) cells within the tissue, as well as lipid droplet diameters and areas of ML adipocytes *P<0.05 versus Eu; #P<0.05 versus Hypo.

Histological analysis of BAT showed that in Hypo animals, the parenchyma displayed many large multilocular adipocytes that contained lipid droplets of increased size compared to Eu droplets (Fig. 2A,B). Compared to Eu animals, the multilocular adipocyte area in Hypo rats was about 15% greater (Fig. 2C), with a 40% greater lipid droplet diameter (Fig. 2C). In addition, the Hypo parenchyma showed an increased number of unilocular cells (Fig. 2). No difference in multilocular or paucilocular cells was observed between Eu and Hypo BAT parenchyma.

Administration of T2 to Hypo rats significantly reduced the number of unilocular and paucilocular cells, while at the same time increasing the number of multilocular ones (Fig. 2). In T2-treated animals, multilocular brown adipocytes appeared smaller in size than in either Hypo (−29%) or Eu ones (−18%; Fig. 2C). A similar trend was observed when the diameter of lipid droplets in multilocular cells was analyzed (−38% versus Hypo; −13% versus Eu; Fig. 2C). Collectively, the above data suggest that T2 was effective both at maintaining/increasing the phenotype of the multilocular cell-type and, consequently, improving BAT activation.

**Figure 3:**
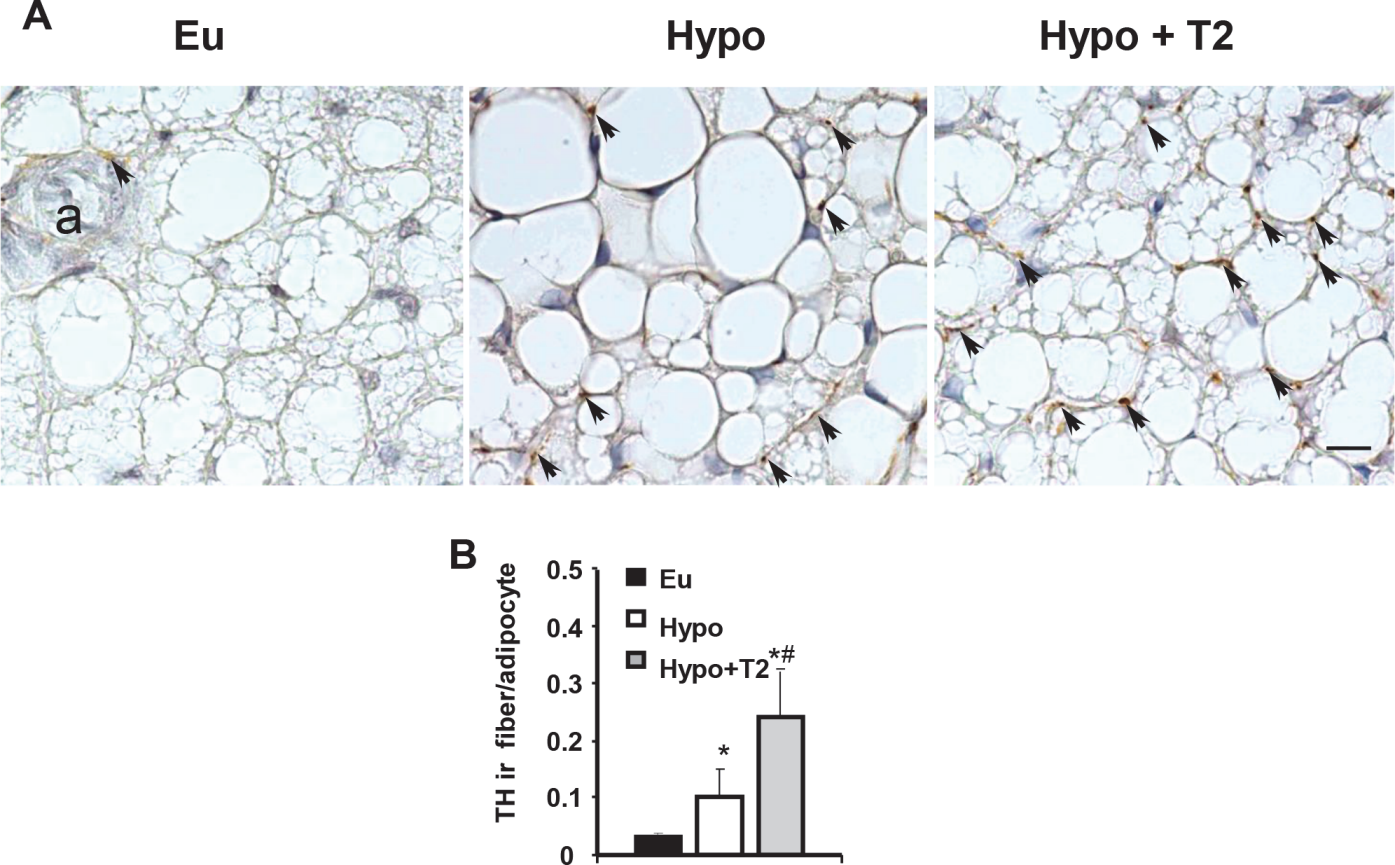
Effects of hypothyroidism and T2 administration to Hypo rats on BAT sympathetic innervation. (A) Parenchymal noradrenergic innervation was detected by immunohistochemistry. Single TH immunoreactive fibers were closely associated with brown adipocytes (arrows) and with a small arteriole (a). Scale bar: 10 μm. (B) Bar chart showing the number of parenchymal TH immunoreactive fibers per adipocyte. *P<0.05 versus Eu; #P<0.05 versus Hypo.

**Figure 4:**
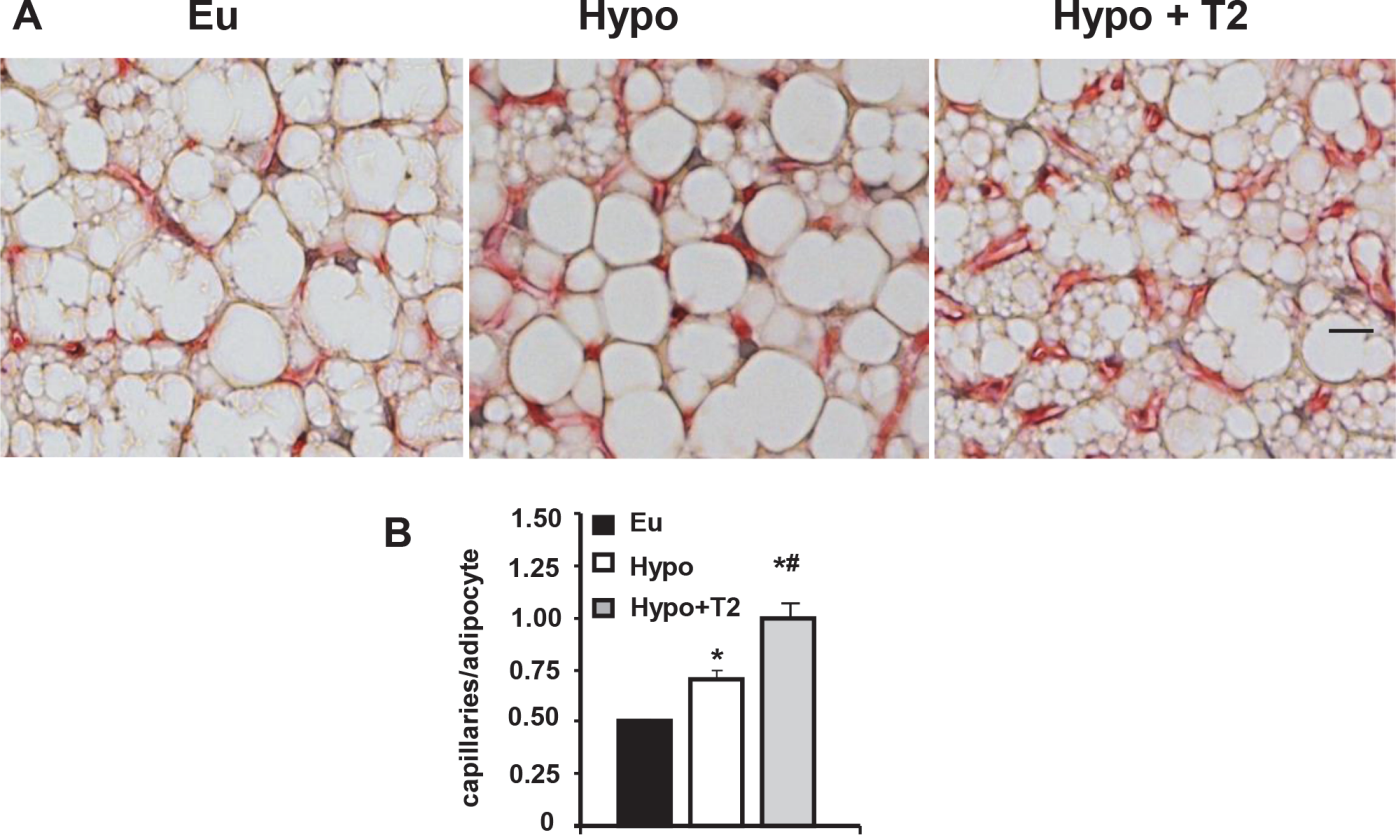
Effects of hypothyroidism and T2 administration to Hypo rats on BAT vascularization. (A) Vascular endothelial cells were visualized by isolectin (BS-1) immunostaining. Scale bar: 10 μm. (B) Bar charts showing the number of BS-1 immunoreactive capillaries per adipocyte. *P<0.05 versus Eu; #P<0.05 versus Hypo.

Sympathetic noradrenergic innervation and capillary vascularization of the tissue were also studied. Examination of TH immunoreactive fibers (a catecholamine-synthesizing enzyme whose expression is related to noradrenergic tone) revealed their density was greater in Hypo compared to Eu rats (+230%; Fig. 3). This effect was enhanced by T2 administration (+140 versus Hypo; Fig. 3). A similar trend was observed when BAT vascularization was analyzed by BS-1 staining of vascular endothelial cells (Fig. 4). Indeed, BAT from Hypo rats exhibited an increase of about 40% in the number of endothelial cells per adipocyte versus Eu BAT, and a further increase in vascularization was observed following T2 administration to hypothyroid rats (+43% versus Hypo; Fig. 4). As a whole, these data indicate that T2 was able to enhance both sympathetic tone and vascularization of BAT.

### BAT functionality

**Figure 5:**
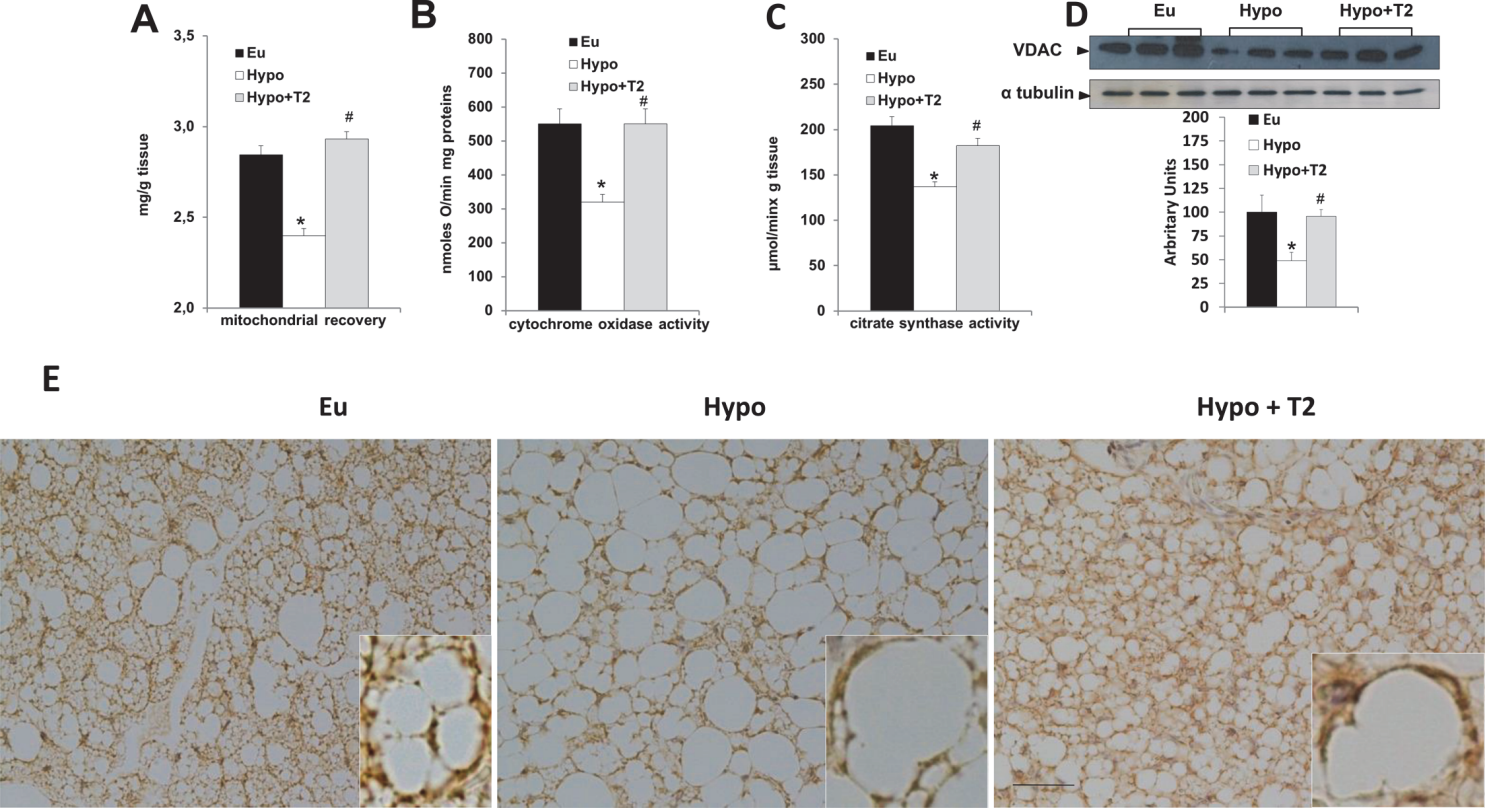
Effects of hypothyroidism and T2 administration to Hypo rats on tissue mitochondrial content and oxidative capacity. (A) Mitochondrial protein/100 mg tissue, (B) cytochrome oxidase (COX) activity, (C) citrate synthase activity, and (D) VDAC1 content detected in whole BAT lysates. Data in the bar chart are represented as means ± standard error of the mean from five independent experiments, each performed in duplicate. *P<0.05 versus Eu; #P<0.05 versus Hypo. (E) Representative COX IV immunoreactivity in BAT. Scale bar: 40 μm.

To assess whether hypothyroidism and T2 administration to Hypo rats might affect BAT mitochondrial density, we evaluated: mitochondrial content (expressed as mitochondrial protein/100 mg tissue), COX activity, citrate synthase, VDAC1 content and COX IV immunohistochemistry (Fig. 5). Hypothyroidism and T2 administration to Hypo rats had opposite effects on mitochondrial BAT content (Fig. 5A). In fact, hypothyroidism reduced mitochondrial content by about 24% (versus Eu), while T2 administration significantly enhanced it (+35% versus Hypo; Fig. 5A). Next, we looked at the COX activity in BAT homogenates. Hypothyroidism significantly reduced COX activity (−40% versus Eu), while T2 administration increased its activity (+65% versus Hypo), restoring it to values observed in tissues from Eu rats (Fig. 5B). These data are in line with the detection of citrate synthase activity (Fig. 5C) and VDAC1 content in BAT lysates (Fig. 5D), wherein they were reduced by 33% and 50%, respectively, in Hypo versus Eu lysates; T2 administration to hypothyroid rats restored these levels to those of Eu lysates.

The above data are also in accordance with our immunohistochemical examination of COX IV, a marker of mitochondrial content (Fig. 5E). Indeed, COX IV immunoreactivity was reduced in BAT from Hypo rats, and administration of T2 to Hypo rats significantly enhanced it. COX IV immunoreactivity was evident in the cytoplasm of multilocular cells, but many paucilocular and unilocular showed strong COX IV immunoreactivity, as well (Fig. 5E). Likewise, many unilocular adipocytes in the peripheral region of BAT depots from T2-treated rats displayed strong COX IV immunoreactivity (data not shown).

**Figure 6:**
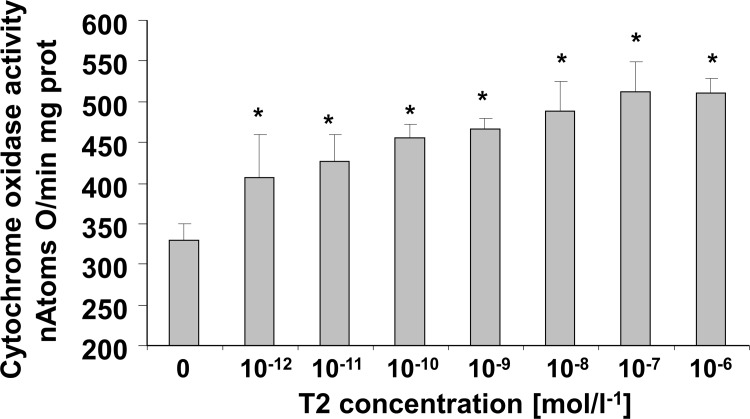
Dose-dependent effect of in vitro addition of T2 on cytochrome oxidase (COX) activity in Hypo BAT homogenates. Values represented means ± standard error of the mean from five independent experiments, each performed in duplicate. *P<0.05 versus 0 M.

To determine if T2 also directly influences the oxidative capacity of the tissue (measured as COX activity), we evaluated its effect when added *in vitro* to BAT homogenates obtained from Hypo rats (Fig. 6). A dose-response relationship between T2 concentration and COX activity was analyzed. We found that a significant stimulatory effect was already evident at pM concentrations of T2. The maximum stimulatory effect of T2 on COX activity (+50% versus 0 M) was obtained when the concentration of T2 reached about 10^−8^ M, while a half-maximal increase was achieved at a concentration of about 10^−11^ M (Fig. 6).

**Figure 7:**
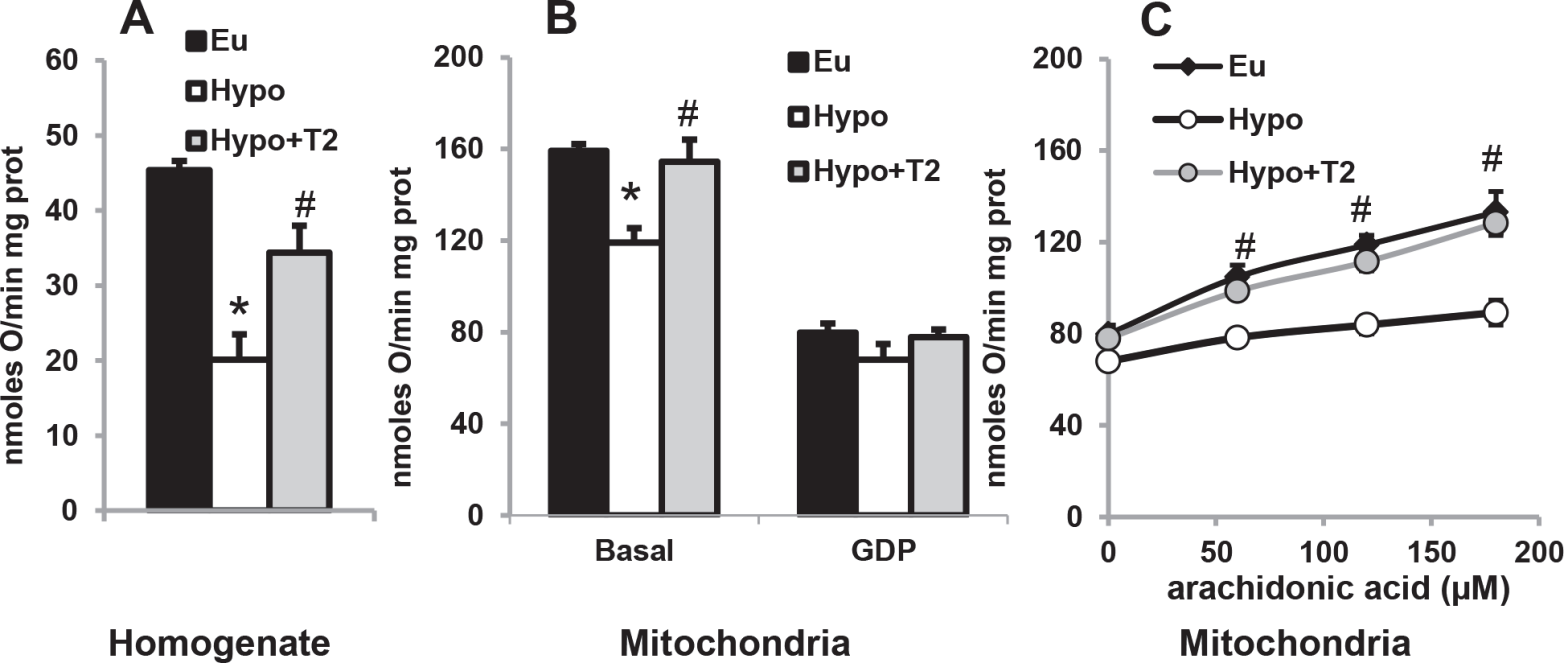
Effects of hypothyroidism and T2 administration to Hypo rats on mitochondrial respiration rate. (A) Mitochondrial respiration evaluated in whole homogenates energized with α-glycerophosphate. (B) Mitochondrial respiration evaluated in isolated mitochondria in the absence and presence of 1 mM GDP. (C) An increase in oxygen consumption rate caused by addition of arachidonic acid to GDP inhibited-mitochondria. Values represented as means ± standard error of the mean from five independent experiments, each performed in duplicate. *P<0.05 versus Eu; #P<0.05 versus Hypo.

We next evaluated mitochondrial respiration rate (which in BAT mitochondria is an index of thermogenesis since it is uncoupled from ATP synthesis) both in the whole homogenate (Fig. 7A) and in isolated mitochondria (Fig. 7B,C). When detected in the whole homogenate, mitochondrial α-glycerophosphate-energized respiration was reduced in BAT obtained from Hypo animals (−65% versus Eu), while T2 administration significantly enhanced it (+70% versus Hypo; Fig. 7A). A similar trend was observed when the respiration rate was examined in isolated mitochondria, although the differences were smaller: a 25% decrease in respiration rate was observed in Hypo mitochondria compared to Eu ones, and an increase of about 30% was observed following T2 administration to hypothyroid rats (Fig. 7B).

**Figure 8:**
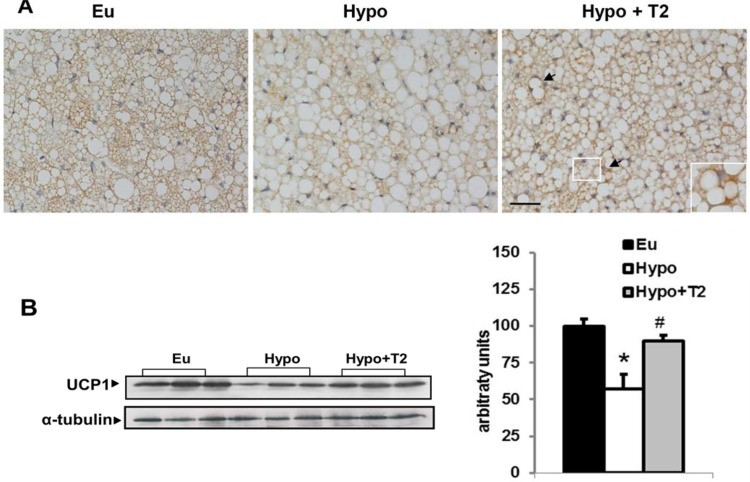
Effects of hypothyroidism and of T2 administration to hypothyroid rats on BAT UCP1 content. (A) Immunohistochemistry for UCP1 in representative sections of BAT. Inset: enlargement of the framed area showing an intensely stained PL cell (arrow). Scale bar represents 40 μm. (B) Representative western blots and quantification of the signals of UCP1 protein levels detected in the whole lysate and at the mitochondrial levels. For total lysate, each lane contained 15 μg of protein from a single rat. Bar charts show quantifications of the signals (expressed relative to the value for the euthyroid controls obtained from 5 rats). *P<0,05 vs Eu, #P<0,05 vs hypo

In experiments in which GDP, a UCP1 inhibitor, (7B) and increasing amounts of arachidonic acid (which removes GDP inhibition of UCP1) were sequentially added to energized mitochondria (Fig. 7C), UCP1 activity seemed to underlie the differences in isolated mitochondrial respiration rates. GDP significantly inhibited the respiration rate in all groups considered, and abolished the differences in respiration rate observed between groups (7B). Following sequential addition of arachidonic acid to GDP-inhibited mitochondria, differences between groups were restored (Fig. 7C). When arachidonic acid reached a concentration of 180 μM, increases in respiration rate were observed in mitochondria from Eu (67%), Hypo (31%), and Hypo+T2 rats (62%), respectively (all versus 0 μM; Fig. 7C). These data indicate that mitochondria from Hypo rats have the lowest sensitivity to fatty acid-induced thermogenesis. These results are in accordance with UCP1 immunoreactivity in whole tissues and UCP1 protein content detected by Western blot (Fig. 8). Indeed, BAT from Hypo rats displayed weak UCP1 staining, whereas T2 administration increased UCP1 in all multilocular cells of brown adipose parenchyma; interestingly, some paucilocular adipocytes showed strong labelling (Fig. 8A). In addition, Western blotting (8 B) indicated that hypothyroidism significantly decreased mitochondrial UCP1 levels, while T2 administration significantly increased it.

### Effect of hypothyroidism and T2 administration to hypothyroid rats on BAT PGC-1α protein levels

**Figure 9:**
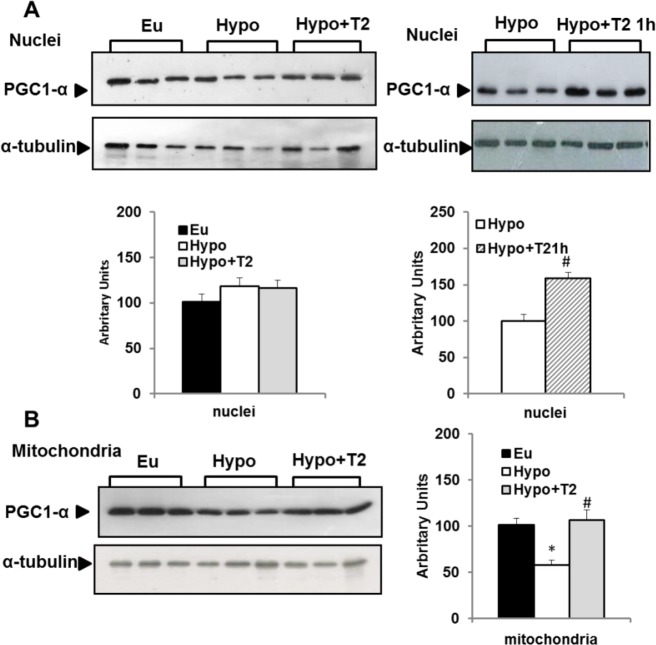
Effects of hypothyroidism and of T2 administration to Hypo rats on nuclear and mitochondrial PGC1-α content. Representative western blots and quantification of the signals are shown. Each lane contained 15 μg of protein from a single rat. Bar charts show quantifications of the signals (expressed relative to the value for the Euthyroid controls obtained from 5 rats). Values represent mean ± SE * P <0.05 vs. Eu. #P<0.05 vs. Hypo.

In view of the crucial role played by PGC-1α in BAT recruitment, we evaluated its protein content in both nuclei- and mitochondrial-enriched fractions by Western blot. When compared to Eu rats, hypothyroidism did not affect nuclear levels of PGC-1α, but did significantly decrease the amount of protein detected in the mitochondria-enriched fraction (Fig. 9). Chronic administration of T2 to hypothyroid rats, on the other hand, did not affect BAT nuclear levels of PGC-1α. (Fig. 9), but induced a significant increase in mitochondrial PGC-1α levels, thus restoring it to Eu levels (Fig. 9). However, to test the possibility that T2 might promote a transient increase in the nuclear level of PGC-1α that was no longer evident after 1 week of T2 treatment, we also evaluated PGC-1α nuclear content in BAT from Hypo+ T21h rats. Indeed, within 1 h of injection, T2 enhanced PGC-1α nuclear content, suggesting that this iodothyronine promotes a rapid, but transient, increase in the nuclear content of PGC-1α.

### Effect of T3 administration to hypothyroid rat on BAT thermogenic parameters

**Figure 10:**
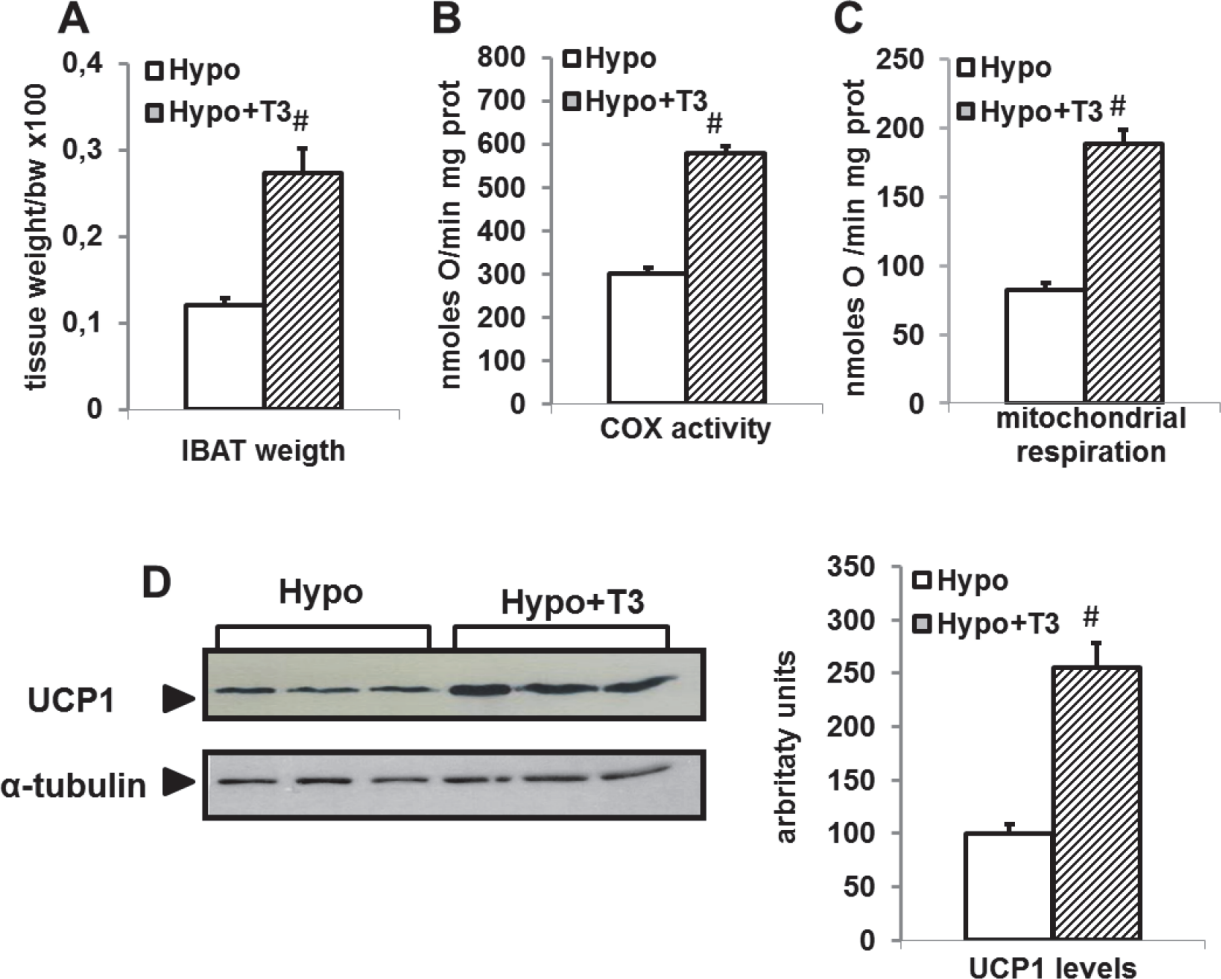
Effects of T3 administration to Hypo rats on (A) BAT weight, (B) Cytochrome oxidase (COX) activity, (C) mitochondrial respiration, and (D) UCP1 content in Hypo rats. (D) Representative Western blots and histogram of UCP1 protein levels in whole lysates (15 μg of protein/rat/lane). The bar charts shows blot signal quantification relative to Eu (n = 5 rats). *P<0.05 versus Hypo.

**Figure 11:**
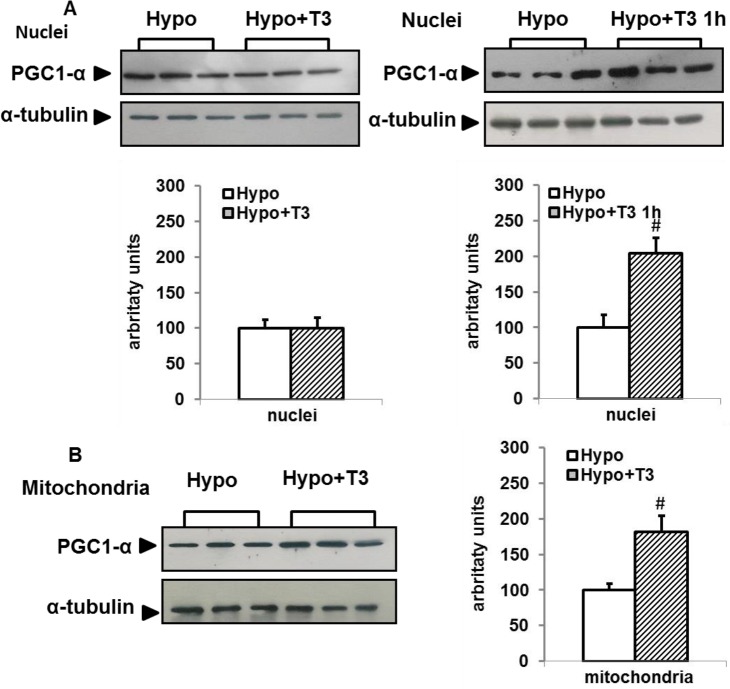
Effects of T3 administration to Hypo rats on nuclear and mitochondrial PGC1-α content. Representative Western blots and respective histograms of respective lysates. Each lane was loaded with 15 μg protein from a single rat. The bar charts show blot signal quantification relative to Hypo (n = 5 rats). Values represent mean ± standard error of the mean. #P<0.05 versus hypo

We next investigated the possibility that some of the molecular events underlying the effect of T2 on BAT thermogenesis could be also activated by T3. Thus, we first confirmed the ability of T3 to activate BAT thermogenesis when administered to Hypo rats by detecting its capacity to increase BAT mass, COX activity, mitochondrial respiration, and UCP1 levels. As reported in Fig. 10, administration of T3 to Hypo rats induced a significant increase in BAT mass (+200%), COX activity (+93%), UCP1 levels (+150%), and mitochondrial respiration (+70%), confirming the well-known responsiveness of BAT thermogenesis to T3 treatment. We next looked at the effect exerted by T3 on PCG-1α, which was very similar to that induced by T2. In fact, administration of T3 to Hypo rats induced a rapid and transient increase in nuclear levels of PGC-1α (Fig. 11). Indeed, in Hypo+T31h rats, T3 promoted a rapid increase of PGC-1α (+100%) that was not evident after 1 week of T3 treatment. At the mitochondrial level an increase of PGC-1α (+80%) was observed following 1 week of T3 administration.

## DISCUSSION

Current attempts to counteract metabolic dysfunction focus on thyroid hormone mimetics that selectively increase energy expenditure [42,43]. In recent years, accumulating evidence has implicated T2 as a “fat burning” agent because of its ability to increase both fatty acid oxidation and metabolic rate. T2 is able to prevent the over-weight and some related metabolic disorders induced by a high-fat diet [28,30–32], while being devoid of side effects, especially at the cardiac level [28,30–32], as also reported in human [33]. The discovery of functional BAT in adult humans that is involved in energy homeostasis and metabolism has revived interest in this tissue and molecules able to increase its thermogenic capacity. As a main endocrine regulator of BAT [19,44], T3 represents an intriguing metabolic stimulating agent, but dangerous side effects exclude its pharmacological use. So far, no data on the ability of thyroid hormone derivatives to activate BAT thermogenesis are present in literature. Here, we present data on the effects of hypothyroidism on BAT thermogenesis and the ability of T2 to activate this tissue.

### Effect of hypothyroidism on BAT thermogenesis

As expected, our data demonstrate that mitochondria play a significant role in the metabolic variations induced by different thyroid status. Indeed, we found that BAT from Hypo rats had reduced maximal oxidative capacity and reduced mitochondrial thermogenesis (compared to the Eu condition). These phenomena presumably result from concomitant reductions in mitochondrial numbers and respiration rate exemplified by the reduced mitochondrial protein content/100 mg tissue, citrate synthase activity, VDAC1 content, and reduced immunohistochemical detection of COX IV. In addition, these results implicate UCP1 as one of the factors underlying impairment of BAT mitochondrial thermogenesis in hypothyroidism. In particular, our data reveal reduced UCP1 immunoreactivity/levels in Hypo tissue, abolition of the difference in respiratory rate between Eu and Hypo mitochondria by GDP, and a reduced ability of arachidonic acid to reactivate GDP-inhibited respiration in Hypo mitochondria. In agreement with these results, our morphological analysis revealed that in the hypothyroid state, brown adipocytes are less active. Indeed, Hypo rats had more lipid-replete BAT adipocytes, characterized by an increased number of unilocular adipocytes, which are akin to white adipocytes.

### Effects of T2 administration to hypothyroid rats

Administration of T2 to Hypo rats activated BAT thermogenesis, as supported by our functional and morphological data. Indeed, at the functional level, T2 increased whole homogenate COX activity, known to be an index of the maximal oxidative capacity of a tissue. It should be noted that in addition to transferring electrons to oxygen molecules, the COX IV complex translocates protons across the mitochondrial membrane, thereby helping to establish the proton electrochemical gradient. In BAT, however, a significant part of this potential is dissipated as heat instead of being used to synthesize ATP. This occurs because of the presence of UCP1; thus, activation of COX activity would enhance mitochondrial thermogenesis.

The effect of T2 on COX activity, at least in part, would seem to be independent of sympathetic activation and directed at mitochondrial level, since it was observed not only following *in vivo* administration of T2, but also following its *in vitro* addition to BAT homogenates obtained from Hypo rats. These data are in accordance with the ability of T2 to interact directly with the COX complex, thereby promoting its activation [45,46]. Apparently, UCP1 also mediates the effect of T2 on mitochondrial BAT thermogenesis since T2 administration enhanced UCP1 immunopositivity/levels in multilocular cells. In addition, inhibition of mitochondrial thermogenesis by GDP and its reactivation by arachidonic acid were both greater in mitochondria from T2-treated Hypo rats than in those from Hypo ones. Moreover, we found that the ability of T2 to activate BAT thermogenesis is associated with changes in adipocyte morphology. Indeed, T2 administration reversed the “white-like” appearance of brown adipocytes induced by hypothyroidism by enhancing the percentage of multilocular cells within the tissue and reducing that of unilocular ones, decreasing the lipid droplet diameter, and increasing the mitochondrial contents.

Sympathetic activation was also associated with the effects of T2 on BAT in this study. However, contrary to results observed for other parameters considered, administration of T2 does not restore sympathetic innervation to Eu levels, but rather further increases the number of noradrenergic positive fibers per adipocyte which are already increased by hypothyroidism. Considering the effects exerted by T2 at the cellular level, stimulation of the sympathetic pathway was probable. Unexpectedly, sympathetic activation was observed in the hypothyroid state. However, this observation was in agreement with previous reports showing that in the hypothyroid state, generally at cellular and tissue levels, responses to catecholamines are reduced, whereas the central sympathetic output reaching the tissues is enhanced [47]. Such a process also occurs in BAT. In fact, the thermal response of BAT to norepinephrine administration has been shown to be drastically reduced in hypothyroid mice compared with euthyroid ones [48]. Moreover, brown adipocytes freshly isolated from hypothyroid animals were shown to be adrenergically desensitized [49, 50]. As the classical function of BAT is to generate heat for thermoregulatory purposes, the enhanced sympathetic innervation and vascularization observed in Hypo versus Eu rats in the present study could be interpreted as a form of compensation for the impaired capacity of BAT to produce heat. Higher BAT vascularization would facilitate distribution of the heat produced that is depressed during hypothyroidism.

By promoting sympathetic tone activation, administration of T2 to hypothyroid rats would lead to an increase in parenchymal brown adipocytes, which are well innervated and vascularized. Plausibly, sympathetic activation, which is known to promote induction of angiogenesis, could also be responsible for the heightened BAT vascularization induced by T2, which would permit an increase in blood supply needed to support the higher oxygen and substrate requirement during BAT recruitment [51]. The present study, however, does not allow us to determine to what extent the sympathetic nervous system is involved in recruitment of BAT thermogenesis by injection of T2, and despite this aspect is intriguing, at the moment is out of the scope of manuscript. Further detailed studies will be needed to clarify this aspect.

Taken as a whole, data reported herein indicate that BAT may underlie part of the observed effect of T2 on rat energy expenditure. However, although T2 administration normalized most of the thermogenic parameters depressed by hypothyroidism (i.e., mitochondrial content, respiration, maximal oxidative capacity, etc.), it did not normalize rat energy expenditure. This discrepancy is likely because each organ of the body contributes to the total energy expenditure, and thus the thermogenic effect of T2 on metabolically active organs and tissues other than BAT must be considered. For example, we recently reported that one of the main contributors to rat energy expenditure in skeletal muscle, mitochondrial proton conductance (index of mitochondrial thermogenesis), is reduced by hypothyroidism and that 1 week administration of T2 significantly enhanced its levels but did not restore it to Eu levels [27].

The transcriptional co-activator PGC-1α is considered as a major regulator of brown adipocyte function and is one of the main players in the activation of BAT thermogenesis. PGC-1α has been also implicated as a central regulator of mitochondrial gene expression and as an essential component of mitochondrial biogenesis [2]. Indeed, nuclear PGC-1α coactivates nuclear respiratory factors-1 and -2, which regulate expression of mitochondrial transcription factor A (mtTFA), a nuclear-encoded transcription factor essential for replication, maintenance, and transcription of mitochondrial DNA (mtDNA) [2]. In addition, mitochondrial PGC-1α localization has recently been described [53]. In mitochondria, PGC-1α is associated with nucleoids and forms a multiprotein complex with mtTFA at the mtDNA transcription start site, thus supporting the putative role of mitochondrial PGC-1α as a transcriptional coactivator of mtTFA [54].

Data reported in the present study indicate that PGC-1α may mediate the effect of T2 on BAT activation since an increase in PGC 1α protein levels was observed both in the nucleus (at 1 h) and in mitochondria (after 1 week) in Hypo+T2 rats. This suggests T2 may activate coordinate nuclear and mitochondrial processes that ultimately promote mitochondrial biogenesis and BAT thermogenesis. Moreover, the enhancing effect of T2 on the nuclear levels of PGC-1α was transient (Fig. 9), suggesting that this coactivator is involved only in the first phase of nuclear events triggering metabolic adaptation in BAT. The effect exerted by T2 on BAT PGC-1α seems to mimic that induced by T3. Even though our purpose was only to determine if T2 and T3 triggered similar mechanisms to activate BAT thermogenesis, data reported herein give novel insight into the effect exerted by T3 on PGC-1α protein levels in BAT. To our knowledge, this is the first report to describe a rapid and transient increase in PGC-1α BAT nuclear levels following *in vivo* administration of T3, and the ability of T3 to increase PGC-1α levels in mitochondria.

Consideration of the molecular events underlying the effect of T2 on BAT thermogenesis should also encompass recent evidence showing the ability of T2 to specifically bind a long thyroid hormone receptor β1 isoform (L-TRβ1) in Tilapia (a teleost fish species) [55]. L-TRβ1 contains a nine amino acid insert at the beginning of the ligand domain. While T2 only interacts with this isoform and activates L-TRβ1 in a cell- and promoter-specific manner [55], T3 preferentially binds the short isoform that lacks this insert. Both T3 and T2 participate in Tilapia growth processes, and their effects are mediated by different specific TRβ1 isoforms, supporting the notion that T2 plays a physiological role in this species [56]. However, no evidences exists suggesting the interaction between T2 and TRβ1 affects energy metabolism in Tilapia or other species.

Quite recently Jones et al [57], by using diet-induced obese mice as animal model, observed thyromimetic effects of T2 on mice body composition and energy metabolism, thus confirming some of our previous data concerning the effects induced by T2 on high fat fed rats. On the other hand, the same authors [57] also observed thyromimetic effects of T2 on hypothalamus-pituitary-thyroid axis, hepatic and pituitary gene expression, and heart weight, thus suggesting the involvement of TRs in the effects induced by T2. However, several differences exist between the experimental design used by Jonas et al and the one reported in the present manuscript: animal strain, animal housing temperature, diet, dose of T2 administered, duration of treatment. These wide differences, crucial for metabolic studies, could explain the results and conclusion’s dissimilarity, when compared to our report.

In particular, the doses of T2 used in the study of Jones et al [57], were unusually high; clear cut effects of T2 were observed only when it was chronically administered to mice at a dose of 250 μg/100 g bw. When using this very high dose, unspecific effects may be elicited, and the interaction of T2 with TRs could be facilitated, despite the much lower affinity of TRs for T2 compared to T3. On the other hand, we [26–31] and other groups [52, 58–61] reported that, T2 chronically administered to rats at a dose of 25 μg/100 g bw was able to induce metabolic effects; moreover it was devoided of side effects on heart mass (present paper and refs [28, 30, 31, 58]), heart rate [28, 61] and TSH levels [58]. Thus confirming the importance of the dose of T2 and of the animal strain used for the obtained results and the conclusion that can be drawn.

Alternatively, there are studies which suggest some T2 effects are independent of TRs. For example, T2 has been shown to weakly transactivate TRβ and TRα target genes in different *in vitro* and *in vivo* systems [30,62–63]. We have also previously reported that the effect of an *in vivo* administration of a single dose of T2 on hypothyroid rat resting metabolism is independent of *de novo* transcription [29,36]. Furthermore, the present study shows that *in vivo* T2 administration to hypothyroid rats promotes rapid nuclear translocation of PGC-1α and that *in vitro* addition of T2 to Hypo BAT homogenates rapidly stimulates COX activity. However, although the last described results indicate a possible TR-independent mechanism(s) of T2 action, we cannot exclude that the long term effects observed in this study could be the result of T2 binding to TRs.

As a whole, despite the possibility that part of the observed effects exerted by T2 on BAT thermogenesis, here reported, can be mediated by the binding of T2 to TR, only systematic studies (likely on TR null mice) will allow to definitively clarify this aspect. Thus, further experimentation is required to clarify this point.

Another point to consider when administering iodothyronines *in vivo* is their possible conversion to metabolites with biological activity. For instance, T3 can be converted to T2 [36], while T2 can be further converted to monoiodo-L-tyrosine, iodothyronamine (T1AM), or 3,5-diiodothyroacetic acid (DIAC). In the present study, we used a validated rat model of severe hypothyroidism and inhibition of the three known types of deiodinase enzymes [29,36] to ensure our observations were due to the injected iodothyronines (T2 or T3) rather than their deiodinated products or conversion of T3 to T2. Furthermore, involvement of T1AM is unlikely as it produces a hypometabolic state and reduces body temperature in rodents [64–65]. However we cannot discount the possibility that deamination of T2 to DIAC took place in our animal model, but the metabolic effects of DIAC on thermogenesis have not been described thus far.

Overall, the results of the current study other than contributing to elucidate the hormonal control of energy homeostasis, could also be of clinical interest. Indeed, the discovery of functional BAT in adult humans, its significant involvement in energy metabolism [6,18], and potential control of triglyceride clearance and glucose disposal [10,11,13,18] suggest that T2 may be able to counteract metabolic dysfunction and related diseases in humans. In addition, T2 may also help facilitate weight loss during caloric restriction (i.e., dieting). Evolutionarily, caloric restriction causes a reduction in T3 levels that prevents further weight loss. Since T2, contrary to T3, is not associated with thyrotoxicity or undesirable cardiovascular side effects [28,30,33,61], T2 supplementation while dieting could increase weight loss, when clinically necessary.
